# Supplementary material for: Fairness matters for change: A multilevel study on organizational change fairness, proactive motivation, and change-oriented OCB
Source: PLoS One. 2024 Oct 31;19(10):e0312886. doi: 10.1371/journal.pone.0312886 (PMC11527186; doi:10.1371/journal.pone.0312886)
Supplement: Attachment — Submitted filename: rebuttal letter.docx [file pone.0312886.s001.docx]

August 12, 2024

Dear Dr. Chunyu Zhang and two Outstanding Reviewers:

I am pleased to submit the revised version of our manuscript titled “Fairness Matters for Change: A Multilevel Study on Organizational Change Fairness, Proactive Motivation, and Change-Oriented OCB” (Manuscript ID: PONE-D-24-21250) for consideration for publication in *PLOS ONE*. We are grateful for the detailed and constructive feedback provided by you and the reviewers. We have carefully considered each comment and made substantial revisions to improve the quality and clarity of our manuscript.

Below, we outline the changes made in response to the reviewers’ comments:

- **Abstract Modification**: Following Reviewer 1's suggestion, we have revised our abstract to include information on the origin, methodology, conclusion, and implications of our study, making it more comprehensive and informative.
- **Motivation and Significance**: We have enhanced the introduction and literature review sections to better emphasize the importance of the research problem and our study’s motivation. Additional relevant arguments have been added to highlight the significance of our research questions.
- **Data Analysis and Results Presentation**: In the results section, we have included tables that were previously omitted to present our findings more clearly. Furthermore, we have added the results of the common method bias test to ensure the robustness of our conclusions.
- **Discussion**: We have expanded the discussion section to provide a deeper analysis of the theoretical relationship between change fairness and change-oriented OCB, as well as the practical implications of our findings.
- **References**: We have updated our reference list by incorporating recent relevant literature and removing outdated sources, ensuring that our study is grounded in the most current research.

We believe these revisions have significantly improved the manuscript, and we hope it now meets your expectations. Please find the revised manuscript and a detailed point-by-point response to the reviewers’ comments attached. Changes to the manuscript have been highlighted in red within the main text of the paper. Thank you for your consideration of our revised manuscript. We look forward to your positive response.

Sincerely,

Bin Ling, Qu Yao, Yutong Liu, Dusheng Chen

1. When submitting your revision, we need you to address these additional requirements.

Please ensure that your manuscript meets PLOS ONE's style requirements, including those for file naming. The PLOS ONE style templates can be found at

https://journals.plos.org/plosone/s/file?id=wjVg/PLOSOne_formatting_sample_main_body.pdf and

https://journals.plos.org/plosone/s/file?id=ba62/PLOSOne_formatting_sample_title_authors_affiliations.pdf

**Response：**

We have reviewed and revised our manuscript to ensure it meets PLOS ONE’s style requirements.

2. Thank you for stating in your Funding Statement:

This research was supported by Soft Science Research Plan Project for Nanjing (202303001)

Please provide an amended statement that declares all the funding or sources of support (whether external or internal to your organization) received during this study, as detailed online in our guide for authors at http://journals.plos.org/plosone/s/submit-now. Please also include the statement “There was no additional external funding received for this study.” in your updated Funding Statement.

Please include your amended Funding Statement within your cover letter. We will change the online submission form on your behalf.

**Response:**

Thank you for your comment. We have amended the Funding Statement to declare all sources of support received during this study. The updated statement is as follows:

*Amended Funding Statement: This research was supported by Soft Science Research Plan Project for Nanjing (202303001). There was no additional external funding received for this study*.

We have included this amended Funding Statement in our cover letter and will ensure it is reflected accurately in the manuscript.

3. In the online submission form, you indicated that the datasets analyzed during the current study are available from the corresponding author on reasonable request.

All PLOS journals now require all data underlying the findings described in their manuscript to be freely available to other researchers, either a. In a public repository, b. Within the manuscript itself, or c. Uploaded as supplementary information.

This policy applies to all data except where public deposition would breach compliance with the protocol approved by your research ethics board. If your data cannot be made publicly available for ethical or legal reasons (e.g., public availability would compromise patient privacy), please explain your reasons on resubmission and your exemption request will be escalated for approval.

**Response:**

We have made the datasets from our study freely available in a public repository. The data can be accessed at the following link: https://osf.io/5xcun/?view_only=d29a262523c2444ab024d224f4e33579. This link has been added to the Participants and Procedure section.

4. PLOS requires an ORCID iD for the corresponding author in Editorial Manager on papers submitted after December 6th, 2016. Please ensure that you have an ORCID iD and that it is validated in Editorial Manager. To do this, go to ‘Update my Information’ (in the upper left-hand corner of the main menu), and click on the Fetch/Validate link next to the ORCID field. This will take you to the ORCID site and allow you to create a new iD or authenticate a pre-existing iD in Editorial Manager. Please see the following video for instructions on linking an ORCID iD to your Editorial Manager account: https://www.youtube.com/watch?v=_xcclfuvtxQ

**Response:**

Yes, I have. I have validated my ORCID iD in Editorial Manager.

5. We note you have included a table to which you do not refer in the text of your manuscript. Please ensure that you refer to Tables 1, 2, 3 and 4 in your text; if accepted, production will need this reference to link the reader to the Table.

**Response:**

We have reviewed our manuscript and ensured that all tables are referenced in the text.

6. Please ensure that you include a title page within your main document. We do appreciate that you have a title page document uploaded as a separate file, however, as per our author guidelines (http://journals.plos.org/plosone/s/submission-guidelines#loc-title-page) we do require this to be part of the manuscript file itself and not uploaded separately.

Could you therefore please include the title page into the beginning of your manuscript file itself, listing all authors and affiliations.

**Response:**

We have revised our manuscript to include the title page at the beginning of the main document, as per the author guidelines. The title page now lists all authors and their affiliations within the manuscript file itself.

**Response to Reviewer 1:**

**Q1:** The abstract needs to be expanded upon and improved. Crucial information on the origin, methodology, conclusion, and implication needs to be included.

**Response:**

Thank you for your valuable feedback on our manuscript. We have made the following revisions in response to your suggestion to expand and improve the abstract:

**Origin:** At the beginning of the abstract, we have clarified the background and motivation of the study, highlighting the importance of achieving consensus on fairness within the team for the success of organizational change.

**Methodology:** We have detailed the theoretical foundation of the study, specifically the proactive motivation model, and the establishment of a multilevel framework. Additionally, we have mentioned the data source, which includes 597 employees from 107 teams across 43 Chinese companies.

**Conclusion:** The abstract now clearly presents the main findings, stating that group-level perceived organizational change fairness significantly predicts employees' change-oriented OCB through organizational change self-efficacy, involvement, and positive emotional experiences.

**Implication:** We have further elaborated on the practical applications and theoretical implications of our findings, emphasizing the importance of these insights for understanding the cross-level determinants influencing change-oriented OCB.

Thank you again for your suggestions, which have helped us to improve the quality of our manuscript. We appreciate any further feedback you may have.

**Changes: Page 1**

**Q2:** In the section of introduction and review, highlight the significance of studying the relationship between fairness, motivation, and OCB in the context of organizational change. The authors need to clearly address the gap as to why this study is essential.

**Response:**

Thank you for your valuable feedback on our study. We have made the following revisions in response to your suggestion to highlight the significance of studying the relationship between fairness, motivation, and OCB in the context of organizational change in the introduction and literature review sections:

1. **In the Introduction Section:**

***Emphasizing the Study’s Importance.*** We added descriptions of the critical role of organizational change in modern business practices, particularly how perceptions of fairness impact the success of change (Arnéguy et al., 2018; Khaw et al., 2023; Xu et al., 2016). We emphasized the importance of leaders demonstrating procedural fairness during the change process to enhance employees' acceptance of change (Kim et al., 2023).

***Clarifying the Study’s Significance.*** We elaborated on the importance of understanding how team-level perceptions of organizational change fairness influence employees’ proactive motivational states to promote change-oriented OCB. This perspective contributes to theoretical development and provides practical guidance for management practices.

2. **In the Literature Review Section:**

***Identifying the Research Gap.*** We highlighted the shortcomings of current literature in exploring the relationship between fairness, motivation, and OCB in the context of organizational change. While existing studies have examined these variables individually, few have investigated their interactions and manifestations within a multilevel framework (Choi, 2007; Li et al., 2016; Seppälä et al., 2012).

***Detailing the Study’s Importance.*** In the literature review, we specifically discussed how fairness influences proactive motivational states, which in turn promote change-oriented OCB. We referenced Parker et al. (2010)’s proactive motivation model to explain how the three motivational states—“can do,” “reason to,” and “energized to”—drive individual proactive behaviors (Berg & Kauffeld, 2024; Frese & Fay, 2001). We also discussed how perceptions of fairness stimulate employees’ proactive tendencies, fostering change-oriented OCB by enhancing change self-efficacy, involvement, and positive emotional experiences. Furthermore, we pointed out that these proactive motivational states might be amplified or diminished by the moderating effect of change impact.

We believe these changes will improve the clarity and coherence of our research.

**Changes: Page From 2 to 11**

**Q3:** Result tables are not present in the PDF file of the manuscript.

**Response:** Thank you for pointing out the issue with our manuscript. We have corrected this oversight and have submitted a revised version of the manuscript that includes all the necessary result tables and figures. The specific modifications are as follows:

1. **Table 1: Results of Confirmatory Factor Analysis:**

Includes *χ*^2^/*df*, CFI, TLI, RMSEA, and SRMR values for the hypothesized model and several alternative models.

2. **Table 2: Results of Descriptive Statistics, Correlations, and Aggregation Test:**

Provides means (M), standard deviations (SD), ICC1, ICC2, and ***r_wg_*** values for each variable, as well as correlation coefficients at both the individual and team levels.

3. **Table 3: Results of Hierarchical Linear Modeling:**

Shows the results of the null model, total effect model, and hierarchical analysis of change self-efficacy, change involvement, and positive experience to change.

4. **Table 4: Results for Multilevel Mediation Effects by RMediation:**

Provides mediation effect results, including path *a* estimate, path *a* standard errors, path *b* estimates, path *b* standard errors, indirect effects, and 95% confidence intervals.

These tables are now fully included in the revised manuscript to better support our research findings. We appreciate your attention to this matter and look forward to your further review.

**Changes: Page From 14, 18, 19, 21 to 22**

**Response to Reviewer 2:**

**Q1:** I am very grateful for the opportunity to review this paper on organizational change fairness and change-oriented OCB. The research questions in this paper are very interesting, and I greatly appreciate the theoretical mechanisms of proactive motivation presented in it.

1.The introduction is well-written, but the author needs to more clearly explain why it is necessary to use the proactive motivation framework to interpret the relationship between organizational change fairness and change-oriented OCB. This would help in better understanding the research motivation of this paper.

**Response:**

Thank you for your positive feedback and valuable suggestions on our manuscript. In response to your suggestion to more clearly explain why it is necessary to use the proactive motivation framework to interpret the relationship between organizational change fairness and change-oriented OCB in the introduction, **In the Introduction Section** we have made the following revisions:

1. **Necessity of Using the Proactive Motivation Framework**

We have added a paragraph that explains in detail how the proactive motivation framework systematically elucidates the influence of individual motivational states (“can do,” “reason to,” and “energized to”) on proactive behaviors. These motivational states help better explain why employees, when perceiving fairness in the organizational change process, exhibit higher levels of change-oriented OCB. Specifically, perceived fairness can stimulate employees’ change self-efficacy, involvement, and positive emotional experiences, thus encouraging them to proactively support organizational change.

2. **Theoretical Support**

We have cited Parker et al. (2010) to further illustrate how the proactive motivation framework aids in understanding individuals’ motivation and behavior in the face of change. We also discussed how existing research supports the use of this theoretical framework in explaining the relationship between fairness in organizational change and change-oriented OCB.

We believe these revisions will better clarify the research motivation and theoretical foundation of our study. We appreciate any further feedback.

**Changes: Throughout the manuscript.**

**Q2:** The literature in this paper is outdated; I recommend that the authors supplement it with newer sources.

**Response:** Thank you for pointing out the importance of precision in our choice of terminology. Thank you for your valuable feedback on our manuscript. In response to your comment regarding the outdated literature, we have thoroughly updated our references to include more recent studies, ensuring the content is both current and relevant. The specific changes are as follows:

***We have added the following references***: Berg and Kauffeld (2024); Fullerton et al. (2021); Kalra et al. (2021); Khaw et al. (2023); Liao (2023); Si et al. (2023); Unterhitzenberger and Lawrence (2023). These references provide the latest research findings and theoretical support, enhancing the foundation of our study.

***Simultaneously, we have removed the following outdated references***: Bateman and Crant (1993); Brotheridge (2003); Glomb et al. (2011); Herold et al. (2008); Riolli and Savicki (2006); Roberson (2006); Sagie et al. (1990); Tyler and De Cremer (2005). These references have been replaced by more recent and relevant studies.

With these updates, we have ensured that the references cited are up-to-date and pertinent. We believe these changes will significantly enhance the academic contribution and practical relevance of our paper. Thank you again for your valuable suggestions.

**Changes: Throughout the manuscript.**

**Q3:** On page ten, “employee involvement” should be highlighted as involvement in change. It is recommended to emphasize in the writing that it refers to employees’ involvement in change, not just general employee work involvement.

**Response:** Thank you for your valuable suggestion. We have clarified “employee involvement” as “involvement in change” on page ten and emphasized that it specifically refers to employees’ involvement in change, not just general work involvement.

**Changes: Throughout the manuscript.**

**Q4:** The author uses both single and double quotation marks interchangeably, such as 'can do,' 'reason to,' and 'energized to.' It is recommended to use double quotation marks consistently throughout the text.

**Response:** Thank you for your feedback. We have consistently used double quotation marks throughout the text to ensure uniformity.

**Changes: Throughout the manuscript.**

**Q5:** The paper lacks an analysis of common method bias; it is recommended that the author supplement this analysis.

**Response:** Thank you for your suggestion. We have supplemented the analysis of common method bias (CMB) using Harman’s Single Factor Test and reported the results in the main text.

**Changes: Page 5 and page from 12 to 14**

**Q6:** In the discussion section, the author is advised to deepen the theoretical significance of the relationship between organizational fairness and change OCB. In the practical implications section, enhance the discussion on the significance of the research findings for practical activities, and link them to specific organizational change activities such as change implementation management and change training.

**Response:** Thank you for your valuable feedback on our discussion and practical implications sections. We have made the following revisions based on your suggestions:

In the theoretical implications section, we have deepened the understanding of the relationship between organizational fairness and change-oriented OCB. We extended Parker et al. (2010)'s proactive motivation model by linking group-level organizational change fairness to three proactive states that support change-oriented OCB, emphasizing the importance of organizational change fairness in promoting active engagement in change activities. Additionally, we explored the moderating role of change impact on the perception of fairness, clarifying the interaction between change impact and fairness perception.

In the practical implications section, we enhanced the discussion on the significance of our findings for practical activities and linked them to specific organizational change activities such as change implementation management and change training. We recommend that managers enhance employee identification and commitment to change through regular workshops and training sessions and establish incentive mechanisms to encourage change-oriented behaviors. Furthermore, we highlighted the importance of improving employees' fairness perceptions through transparent communication and employee involvement to support successful organizational change implementation.

**Changes: Page from 22 to 25**

**Q7:** The paper lacks the presentation of four results tables; it is recommended to include results from Table 1 to Table 4.

**Response:** Thank you for your valuable suggestion. We have included the results from Table 1 to Table 4 in the results section of the paper as per your recommendation. For detailed changes, please refer to **Reviewer 1, Q3** (pp. 3-4 in this letter). We believe this modification improves the organization of the manuscript and effectively addresses your concerns.

**Changes: Page From 14, 18, 19, 21 to 22**

**Reference**

Arnéguy, E., Ohana, M., & Stinglhamber, F. (2018). Organizational justice and readiness for change: A concomitant examination of the mediating role of perceived organizational support and identification. *Frontiers in Psychology*, *9*, 1-13. <https://doi.org/10.3389/fpsyg.2018.01172>

Bateman, T. S., & Crant, J. M. (1993). The proactive component of organizational behavior: A measure and correlates. *Journal of Organizational Behavior*, *14*, 103-118. <https://doi.org/10.1002/job.4030140202>

Berg, A.-K., & Kauffeld, S. (2024). Proactive verbal behavior in team meetings: Effects of supportive and critical responses on satisfaction and performance. *Current Psychology*, *43*(23), 20640-20654. <https://doi.org/10.1007/s12144-024-05806-y>

Brotheridge, C. M. (2003). The role of fairness in mediating the effects of voice and justification on stress and other outcomes in a climate of organizational change. *International Journal of Stress Management*, *10*(3), 253-268. <https://doi.org/10.1037/1072-5245.10.3.253>

Choi, J. N. (2007). Change-oriented organizational citizenship behavior: Effects of work environment characteristics and intervening psychological processes. *Journal of Organizational Behavior*, *28*(4), 467-484. <https://doi.org/https://doi.org/10.1002/job.433>

Frese, M., & Fay, D. (2001). Personal initiative: An active performance concept for work in the 21st century. *Research in Organizational Behavior*, *23*, 133-187. <https://doi.org/10.1016/S0191-3085(01)23005-6>

Fullerton, D. J., Zhang, L. M., & Kleitman, S. (2021). An integrative process model of resilience in an academic context: Resilience resources, coping strategies, and positive adaptation. *PLOS ONE*, *16*(2), e0246000. <https://doi.org/10.1371/journal.pone.0246000>

Glomb, T. M., Bhave, D. P., Miner, A. G., & Wall, M. (2011). Doing good, feeling good: Examining the role of organizational citizenship behaviors in changing mood [Article]. *Personnel Psychology*, *64*(1), 191-223. <https://doi.org/10.1111/j.1744-6570.2010.01206.x>

Herold, D. M., Fedor, D. B., Caldwell, S., & Liu, Y. (2008). The effects of transformational and change leadership on employees' commitment to a change: A multilevel study [Article]. *Journal of Applied Psychology*, *93*(2), 346-357. <https://doi.org/10.1037/0021-9010.93.2.346>

Kalra, A., Agnihotri, R., Singh, R., Puri, S., & Kumar, N. (2021). Assessing the drivers and outcomes of behavioral self-leadership. *European Journal of Marketing*, *55*(4), 1227-1257. <https://doi.org/10.1108/EJM-11-2018-0769>

Khaw, K. W., Alnoor, A., Al-Abrrow, H., Tiberius, V., Ganesan, Y., & Atshan, N. A. (2023). Reactions towards organizational change: A systematic literature review. *Current Psychology*, *42*(22), 19137-19160. <https://doi.org/10.1007/s12144-022-03070-6>

Kim, M., Choi, D., Guay, R. P., & Chen, A. (2023). How does fairness promote innovative behavior in organizational change?: The importance of social context. *Applied Psychology*, *73*(3), 1233-1260. <https://doi.org/10.1111/apps.12511>

Li, M., Liu, W., Han, Y., & Zhang, P. (2016). Linking empowering leadership and change-oriented organizational citizenship behavior. *Journal of Organizational Change Management*, *29*(5), 732-750. <https://doi.org/10.1108/JOCM-02-2015-0032>

Liao, P.-Y. (2023). Proactive personality, job crafting, and person-environment fit: Does job autonomy matter? *Current Psychology*, *42*(22), 18959-18970. <https://doi.org/10.1007/s12144-022-03065-3>

Parker, S. K., Bindl, U. K., & Strauss, K. (2010). Making things happen: A model of proactive motivation. *Journal of Management*, *36*(4), 827-856. <https://doi.org/10.1177/0149206310363732>

Riolli, L., & Savicki, V. (2006). Impact of fairness, leadership, and coping on strain, burnout, and turnover in organizational change. *International Journal of Stress Management*, *13*(3), 351-377. <https://doi.org/10.1037/1072-5245.13.3.351>

Roberson, Q. M. (2006). Justice in teams: The activation and role of sensemaking in the emergence of justice climates. *Organizational Behavior and Human Decision Processes*, *100*(2), 177-192. <https://doi.org/10.1016/j.obhdp.2006.02.006>

Sagie, A., Elizur, D., & Koslowsky, M. (1990). Effect of participation in strategic and tactical decisions on acceptance of planned change. *The Journal of Social Psychology*, *130*, 459-465. <https://doi.org/10.1080/00224545.1990.9924612>

Seppälä, T., Lipponen, J., Bardi, A., & Pirttilä-Backman, A.-M. (2012). Change-oriented organizational citizenship behaviour: An interactive product of openness to change values, work unit identification, and sense of power. *Journal of Occupational and Organizational Psychology*, *85*(1), 136-155. <https://doi.org/https://doi.org/10.1111/j.2044-8325.2010.02010.x>

Si, W., Xiao, J., & Chen, L. (2023). Fairly meaningful: Mechanisms linking organizational fairness to perceived meaningfulness. *Journal of Business Ethics*, *187*(1), 53-72. <https://doi.org/10.1007/s10551-022-05271-2>

Tyler, T. R., & De Cremer, D. (2005). Process-based leadership: Fair procedures and reactions to organizational change. *The Leadership Quarterly*, *16*, 529-545. <https://doi.org/10.1016/j.leaqua.2005.06.001>

Unterhitzenberger, C., & Lawrence, K. (2023). Fairness matters: Organisational justice in project contexts. *Production Planning & Control*, 1-16. <https://doi.org/10.1080/09537287.2023.2251424>

Xu, X., Payne, S. C., Horner, M. T., & Alexander, A. L. (2016). Individual difference predictors of perceived organizational change fairness. *Journal of Managerial Psychology*, *31*, 420-433. <https://doi.org/10.1108/JMP-12-2014-0370>
